# Association of obesity and long-term mortality in patients with acute myocardial infarction with and without diabetes mellitus: results from the MONICA/KORA myocardial infarction registry

**DOI:** 10.1186/s12933-015-0189-0

**Published:** 2015-02-18

**Authors:** Miriam Giovanna Colombo, Christa Meisinger, Ute Amann, Margit Heier, Wolfgang von Scheidt, Bernhard Kuch, Annette Peters, Inge Kirchberger

**Affiliations:** Central Hospital of Augsburg, MONICA/KORA Myocardial Infarction Registry, Stenglinstr. 2, 86156 Augsburg, Germany; Helmholtz Zentrum München, German Research Center for Environmental Health (GmbH), Institute of Epidemiology II, Ingolstädter Landstr. 1, 85764 Neuherberg, Germany; Central Hospital of Augsburg, Department of Internal Medicine I–Cardiology, Stenglinstr. 2, 86156 Augsburg, Germany; Hospital of Nördlingen, Department of Internal Medicine/Cardiology, Stoffelsberg 4, 86720 Nördlingen, Germany

**Keywords:** Acute myocardial infarction, Overweight, Obesity, Body mass index, Diabetes mellitus, Long-term mortality, Obesity paradox

## Abstract

**Background:**

Paradoxically, beneficial effects of overweight and obesity on survival have been found in patients after cardiovascular events such as acute myocardial infarction (AMI). This obesity paradox has not been analyzed in AMI patients with diabetes even though their cardiovascular morbidity and mortality is increased compared to their counterparts without diabetes. Therefore, the objective of this long-term study was to analyze the association between body mass index (BMI) and all-cause mortality in AMI patients with and without diabetes mellitus.

**Methods:**

Included in the study were 1190 patients with and 2864 patients without diabetes, aged 28-74 years, recruited from a German population-based AMI registry. Patients were consecutively hospitalized between 1 January 2000 and 31 December 2008 with a first ever AMI and followed up until December 2011. Data collection comprised standardized interviews and chart reviews. To assess the association between BMI and long-term mortality from all causes, Cox proportional hazards models were calculated adjusted for risk factors, co-morbidities, clinical characteristics, in-hospital complications as well as medical and drug treatment.

**Results:**

AMI patients of normal weight (BMI 18.5-24.9 kg/m^2^) had the highest long-term mortality rate both in patients with and without diabetes with 50 deaths per 1000 person years and 26 deaths per 1000 person years, respectively. After adjusting for a selection of covariates, a significant, protective effect of overweight and obesity on all-cause mortality was found in AMI patients without diabetes (overweight: hazard ratio (HR) 0.73, 95% confidence interval (CI) 0.58-0.93; p=0.009; obesity: HR 0.64, 95% CI 0.47-0.87; p=0.004). In contrast, an obesity paradox was not found in AMI patients with diabetes. However, stratified analyses showed survival benefits in overweight AMI patients with diabetes who had been prescribed statins prior to AMI (HR 0.51, 95% CI 0.29-0.89, p=0.018) or four evidence-based medications at hospital-discharge (HR 0.52, 95% CI 0.34-0.80, p=0.003).

**Conclusion:**

In contrast to AMI patients without diabetes, AMI patients with diabetes do not experience a survival benefit from an elevated BMI. To investigate the underlying reasons for these findings, further studies stratifying their samples by diabetes status are needed.

**Electronic supplementary material:**

The online version of this article (doi:10.1186/s12933-015-0189-0) contains supplementary material, which is available to authorized users.

## Background

An elevated body mass index (BMI) is an independent risk factor for increased all-cause mortality in the general population [1]. Furthermore, overweight and obese people are more likely to be affected by cardiovascular events than people of normal weight [2]. However, a controversial survival benefit after cardiovascular events, such as acute myocardial infarction (AMI), has been detected in overweight and obese people [3-8].

Besides excess body weight, diabetes mellitus is a strong risk factor for cardiovascular morbidity and mortality [9,10]. People with diabetes have a significantly increased cardiovascular mortality risk that is even further elevated after an established cardiovascular event [10-12], such as AMI [13]. Furthermore, they show worse short- and long-term outcomes after cardiovascular interventions [14,15] in comparison with people without diabetes. However, only a few studies have examined the association between BMI and long-term mortality after a cardiovascular event separately for patients with and without diabetes and showed inconsistent results [16-18].

Thus, the nature of the obesity paradox in AMI patients cannot be explained thoroughly so far. Additionally, long-term studies controlling for important comorbidities, treatment prior and post AMI as well as in-hospital complications have not been conducted on this topic yet.

Therefore, the objective of this long-term observational study was to analyze the association between BMI and all-cause mortality in patients with and without diabetes using data from an established German myocardial infarction registry and adjusting for a number of relevant risk factors, co-morbidities, clinical characteristics and in-hospital complications as well as treatment measures.

## Methods

As part of the World Health Organization (WHO) project MONICA (Monitoring Trends and Determinants in Cardiovascular disease) the population-based Augsburg Myocardial Infarction (MI) Registry was established in 1984. MONICA was terminated in 1995 and the registry became part of the KORA (Cooperative Health Research in the Region of Augsburg) framework. Since the registry commenced, all cases of coronary death and non-fatal AMI cases of the 25- to 74-year old study population in the city of Augsburg and the two adjacent counties (about 600,000 inhabitants) have been continuously registered. Patients admitted to one of the eight hospitals in the study area and two adjacent counties are included. Methods of case identification, diagnostic classification of events as well as data quality control have been described in detail elsewhere [19,20]. Data collection and follow-up questionnaires of the MONICA/KORA MI registry have been approved by the ethics committee of the Bavarian Medical Association (Bayerische Landesärztekammer) and have been performed in accordance with the Declaration of Helsinki. All study participants gave written informed consent.

### Study population

This study includes all patients consecutively registered between 1 January 2000 and 31 December 2008, who reached the hospital alive and whose survival time exceeded 28 days after AMI. Patients were followed up until December 2011. The data set comprised 5057 patients with a non-fatal first ever AMI aged 28-74 years. Patients with missing data on BMI, diabetes and smoking (n=649) were excluded as well as patients with incomplete data on any of the relevant covariates (n=329). Furthermore, patients who were underweight (BMI < 18.5 kg/m^2^) were not included. The final data set comprised 4054 patients.

Patients excluded from the study sample had a significantly higher crude hazard ratio (HR) for long-term mortality (HR 2.47, 95% confidence interval (CI) 2.17-2.82; p < 0.001) compared with included patients.

### Data collection

Trained study nurses interviewed the study participants during their hospital stay using a standardized questionnaire. The interviews covered demographic information, risk factors, medications prescribed prior to AMI, and co-morbidities. Information on AMI characteristics, medical and drug treatment and in-hospital complications were determined by chart review.

BMI was selected as the index to measure and classify normal weight, overweight and obesity. It was determined by assessment of weight and height during hospital stay and calculated for each patient by dividing the weight (in kilograms) by the square of the height (in meters). According to the WHO, patients are classified as normal weight if their BMI lies between 18.5 and 24.9 kg/m^2^. Overweight patients have a BMI between 25.0 and 29.9 kg/m^2^ and the BMI of obese patients is 30.0 kg/m^2^ or higher [21].

Whether patients were suffering from diabetes mellitus was determined by asking the patients if they had been previously diagnosed with the disease (yes/no). In addition, the information provided by the patients was confirmed by chart review.

Since 2004 a combination of the following four evidence-based medications (EBMs) is considered the standard of care after AMI: anti-platelet agents, beta-blockers, angiotensin-converting enzyme inhibitors (ACEIs) or angiotensin-receptor blockers (ARBs) respectively, as well as statins [22]. Since in-hospital complications, such as cardiogenic shock, occurred infrequently, a variable summarizing the data on all complications available in the data set (cardiac arrest, pulmonary edema, bradycardia, re-infarction, ventricular tachycardia, ventricular fibrillation, cardiogenic shock) was created (yes/no).

To determine all-cause mortality as the outcome of this study, the vital status of the patients in the study population was monitored through the population registries in- and outside the study region until 31 December 2011. The median follow-up time was 6.0 years (IQR 4.1 years).

### Data analysis

Categorical variables were expressed as percentages and continuous variables as mean values with standard deviation (SD). Patients were divided into two groups: patients with and without diabetes. Within the two groups, potential covariates were cross-tabulated with BMI (normal weight, overweight and obesity) as the primary independent variable. Differences in frequencies were tested using Chi^2^ or Fisher’s exact test. To evaluate age differences among the three BMI groups, a one-way ANOVA (analysis of variance) was performed. Kaplan-Meier plots were generated along with bivariate log-rank tests against survival to test for statistical significance.

Cox proportional hazards models were used to examine the association between BMI group and long-term mortality within the two strata (patients with and without diabetes). The proportional hazards assumption (parallel lines of log (-log(event)) versus log of event times) proved to be valid for the majority of the variables except for BMI, age, marital status, education, smoking status, year of infarction, AMI type and ACEIs/ARBs (medication prior infarction). Included as time-dependent covariates, further analyses were made with these variables not complying with the proportional hazards assumption. The results were either not significant and the interaction terms were therefore not included in the analyses or the interaction terms did not make it into the final regression models because they were dropped during the process of backwards selection.

Four Cox proportional hazards regression models were calculated stratified by diabetes (yes/no). First, a crude model was calculated to examine the association between BMI and mortality. Second, a minimal model additionally including the covariates sex and age was calculated. Finally, two parsimonious models, one for patients with diabetes and one for patients without diabetes, were created using backward selection. Variables that made a statistically significant (p < 0.05) contribution were included in the models. The variables sex and age were forced to stay in the models. In order to control for potential cohort effects, we tested whether the year of AMI had an influence on the association between BMI and mortality in both groups, but no effects were found.

Multicollinearity among the independent variables was examined by assessing variance inflation factors (VIF) in the full model prior to backward selection [23].

Interaction effects of sex and age and of BMI group with AMI treatment-related variables were calculated. Significant interaction effects between BMI group and statin treatment prior to AMI as well as prescription of all four EBMs at discharge were found in patients with diabetes. Thus, parsimonious models were separately calculated stratified by statins as medications prior AMI (yes/no) and by receiving all four EBMs at discharge (yes/no).

Finally, parsimonious models were calculated for follow-up periods of one to twelve years in one-year intervals. All data analyses were performed using SAS software, version 9.2 (SAS Institute).

## Results

The study sample consisted of 1190 patients with diabetes (29.4% of the total population) and 2864 patients without diabetes (see Table 1). Both among patients with and without diabetes overweight individuals accounted for the highest percentages with 43.4% (n=516) and 49.3% (n=1411), respectively. In both groups normal weight patients were more likely to have a LVEF < 30%, to receive coronary artery bypass surgery and less likely to receive PCI or any reperfusion treatment compared with overweight or obese individuals. More patients who were overweight or obese than patients who were of normal weight have received prior treatment with ACEIs/ARBs or beta-blockers (only among patients with diabetes). Discharge medication was more common in overweight and obese patients in terms of ACEIs/ARBs, all four EBMs, antiplatelet agents (only in patients with diabetes) and beta-blockers (only in patients without diabetes). Further sample characteristics are presented in Table 1 and Additional file 1.

**Table 1:** **Characteristics of patients with and without diabetes sub-divided into BMI groups (N=4054)**

	**Diabetes (n=1190)**				**No Diabetes (n=2864)**			
	**Normal weight**	**Overweight**	**Obesity**		**Normal weight**	**Overweight**	**Obesity**	
	**BMI 18.5-24.9 kg/m** ^**2**^	**BMI 25-29.9 kg/m** ^**2**^	**BMI ≥ 30 kg/m** ^**2**^		**BMI 18.5-24.9 kg/m** ^**2**^	**BMI 25-29.9 kg/m** ^**2**^	**BMI ≥ 30 kg/m** ^**2**^	
	**(n=226)**	**(n=516)**	**(n=448)**	**p-value**	**(n=848)**	**(n=1411)**	**(n=605)**	**p-value**
**Sociodemographic characteristics**								
Female	65 (28.76)	103 (19.96)	169 (37.72)	<0.0001	247 (29.13)	226 (16.02)	157 (25.95)	<0.0001
Age [years], mean ± SD	63.81 ± 8.79	62.86 ± 8.47	61.96 ± 8.65	0.0262^a^	59.55 ± 10.00	59.56 ± 9.72	58.98 ± 10.06	0.4460^a^
Married^b^	169 (77.17)	390 (77.84)	318 (71.78)	0.0778	602 (72.36)	1084 (77.54)	465 (78.15)	0.0091
Living alone^b^	36 (16.44)	80 (15.97)	99 (22.35)	0.0287	164 (19.71)	212 (15.16)	86 (14.45)	0.0072
School education > 9 years^c^	54 (25.84)	92 (19.49)	90 (20.98)	0.1722	242 (30.67)	405 (30.82)	133 (23.29)	0.0023
**Risk factors and co-morbidities**								
Angina pectoris^d^	52 (23.11)	120 (23.35)	117 (26.29)	0.5028	142 (16.78)	241 (17.10)	124 (20.53)	0.1261
Hypertension	183 (80.97)	448 (86.82)	40.5 (90.40)	0.0026	534 (62.97)	1035 (73.35)	521 (86.12)	<0.0001
Hyperlipidemia	145 (64.16)	382 (74.03)	362 (80.80)	<0.0001	550 (64.86)	1029 (72.93)	455 (75.21)	<0.0001
Stroke	27 (11.95)	51 (9.88)	34 (7.59)	0.1667	43 (5.07)	70 (4.96)	33 (5.45)	0.8981
Smoking								
Current smoker	70 (30.97)	144 (27.91)	116 (25.89)	0.6129	403 (47.52)	533 (37.77)	211 (34.88)	<0.0001
Ex-smoker	77 (34.07)	199 (38.57)	174 (38.84)		200 (23.58)	470 (33.31)	208 (34.38)	
Never smoker	79 (34.96)	173 (33.53)	158 (35.27)		245 (28.89)	408 (28.92)	186 (30.74)	
**Clinical characteristics**								
Re-infarction during hospitalization	30 (13.27)	83 (16.09)	74 (16.52)	0.5252	89 (10.28)	145 (10.04)	59 (9.75)	0.8964
AMI type^e^								
ST-segment elevation MI	81 (36.65)	198 (38.98)	155 (35.07)	0.1190	360 (43.37)	531 (38.23)	227 (38.09)	0.1329
Non-ST-segment elevation MI	118 (53.39)	285 (56.10)	256 (57.92)		434 (52.29)	783 (56.37)	339 (56.88)	
Bundle branch block	22 (9.95)	25 (4.92)	31 (7.01)		36 (4.34)	75 (5.40)	30 (5.03)	
Left ventricular ejection fraction < 30%^f^	35 (21.88)	46 (12.74)	29 (9.93)	0.0015	72 (12.16)	133 (13.34)	33 (7.82)	0.0126
**Diabetes treatment** ^**g**^								
Oral antidiabetic agents	72 (57.60)	182 (63.64)	143 (52.77)	0.0044				
Insulin therapy	40 (32.00)	78 (27.27)	76 (28.04)					
Both antidiabetic agents and insulin therapy	13 (10.40)	26 (9.09)	52 (19.19)					
**Long-term mortality**	66 (29.20)	111 (21.51)	96 (21.43)	0.0453	135 (15.92)	170 (12.05)	61 (10.08)	0.0023

BMI=Body Mass Index, SD=Standard deviation, MI=Myocardial Infarction, AMI=Acute Myocardial Infarction, ACEIs=Angiotensin-converting enzyme inhibitors, ARBs=Angiotensin receptor blockers.

Data are presented as absolute numbers of patients with percentages in brackets, unless stated otherwise.

Chi^2^-Tests were performed to obtain p-values, unless stated otherwise.

^a^p-value was obtained by performing a one-way ANOVA (Analysis of Variance).

^b^n=3988.

^c^n=3784.

^d^n=4043.

^e^n=3986.

^f^n=2388.

^g^n (patients with diabetes)=682.

**Figure 1:**
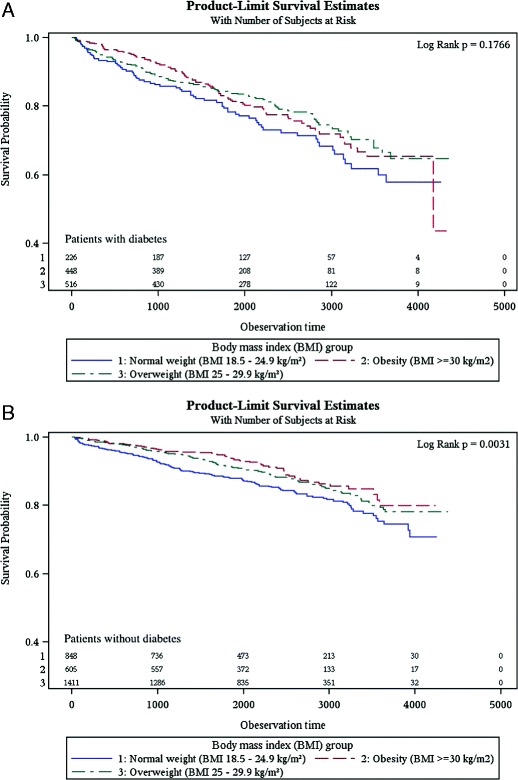
**Kaplan-Meier curves of 12-year survival for normal weight, overweight and obese patients stratified by diabetes status.** Kaplan-Meier curves for **(A)** patients with diabetes and **(B)** patients without diabetes.

Overall, patients with diabetes had a higher long-term mortality rate (40 deaths per 1000 person years; n=273) than patients without diabetes (21 deaths per 1000 person years; n=366). With 50 deaths per 1000 person years (n=66) and 26 deaths per 1000 person years (n=135) normal weight individuals had the highest long-term mortality rate both among patients with and without diabetes, respectively (see Table 1). Furthermore, Kaplan–Meier survival curves demonstrated significant differences in survival between BMI groups in patients without diabetes (p=0.003); in patients with diabetes, however, a difference could not be proven (p=0.177) (see Figure 1).

The unadjusted analyses showed significant protective effects of overweight and obesity in comparison to normal weight on long-term mortality among patients without diabetes; however the same effects could not be proven in patients with diabetes (see Table 2). For overweight and obese patients without diabetes the HRs showed minimal variation even after adjusting for other covariates in the parsimonious models (see Table 2). Overweight patients without diabetes had a statistically significant 0.73-fold risk of dying (95% CI 0.58-0.93; p=0.009) and obese patients without diabetes had an even lower HR of 0.64 (95% CI 0.47-0.87; p=0.004) compared with normal weight patients. In both BMI groups the HRs for patients with diabetes attenuated when covariates were added into the models, however, they remained non-significant.

**Table 2:** **Hazard ratios for mortality associated with elevated body mass index in patients with and without diabetes**

		**No diabetes (n=2864)**		**Diabetes (n=1190)**	
		**HR [95% CI]**	**p-value**	**HR [95% CI]**	**p-value**
Unadjusted model	**Normal weight**	1.0		1.0	
	**Overweight**	0.74 [0.59-0.93]	0.0097	0.76 [0.56-1.02]	0.0707
	**Obesity**	0.62 [0.46-0.85]	0.0023	0.79 [0.58-1.09]	0.1495
Minimal model^a^	**Normal weight**	1.0		1.0	
	**Overweight**	0.72 [0.57-0.90]	0.0040	0.80 [0.59-1.08]	0.1467
	**Obesity**	0.64 [0.47-0.86]	0.0036	0.91 [0.77-1.31]	0.5691
Parsimonious model	**Normal weight**	1.0		1.0	
	**Overweight**	0.73 [0.58-0.93]^b^	0.0087	0.83 [0.61-1.13]^c^	0.2383
	**Obesity**	0.64 [0.47-0.87]^b^	0.0043	0.98 [0.71-1.36]^c^	0.8914

HR=Hazard Ratio, CI=Confidence Interval, BMI=Body Mass Index.

^a^Adjusted for sex and age.

^b^Adjusted for sex, age, stroke, smoking, re-infarction, left ventricular ejection fraction (<30% versus ≥30%), any reperfusion treatment (coronary artery bypass surgery, percutaneous coronary intervention (PCI) or thrombolysis), beta-blockers (medication prior AMI), all four medications at discharge (antiplatelet agents, beta-blockers, ACEIs/ARBs (Angiotensin-converting enzyme inhibitors/Angiotensin receptor blockers), statins).

^c^Adjusted for sex, age, hyperlipidemia, re-infarction, any reperfusion treatment, statins (medication prior AMI), all four medications at discharge.

In the parsimonious models VIF were below 2.5 indicating no relevant multicollinearity among the covariates.

Furthermore, parsimonious models for different observation times (one to twelve years, in 1-year intervals) were calculated comparing HRs of overweight and obese patients with and without diabetes (see Additional files 2 and 3). It could be shown that in AMI patients without diabetes there is a significant protective effect of overweight and obesity on all-cause mortality, which attenuated with increasing observation time while remaining statistically significant. However, in AMI with diabetes being overweight or obese did not result in a survival benefit.

Additionally, parsimonious models were calculated independently stratified by intake of statins prior AMI and number of medications taken at discharge (see Table 3). The stratified analyses resulted in statistically significant HRs of 0.51 (95% CI 0.29-0.89; p=0.0180) and 0.52 (95% CI 0.34-0.80; p=0.0027) for overweight patients with diabetes who received stations prior AMI or all four EBMs at discharge. In contrast, no intake of statins had a beneficial effect on survival of both overweight and obese patients without diabetes, whereas receiving less than four medications at discharge was only beneficial for overweight patients without diabetes. In turn, obese patients without diabetes profited from receiving four medications at discharge.

**Table 3:** **Hazard ratios for mortality associated with elevated body mass index in patients with and without diabetes stratified by prescribed medications before and after myocardial infarction**

		**No diabetes (n=2864)**		**Diabetes (n=1190)**	
		**HR [95% CI]**	**p-value**	**HR [95% CI]**	**p-value**
Statins prior AMI	**Normal weight**	1.0		1.0	
	**Overweight**	0.81 [0.44-1.52]^a^	0.5179	0.51 [0.29-0.89]^b^	0.0180
	**Obesity**	0.59 [0.26-1.35]^a^	0.2115	0.82 [0.46-1.47]^b^	0.5102
No statins prior AMI	**Normal weight**	1.0		1.0	
	**Overweight**	0.71 [0.55-0.92]^a^	0.0082	1.04 [0.71-1.52]^b^	0.8456
	**Obesity**	0.66 [0.47-0.92]^a^	0.0131	1.09 [0.73-1.64]^b^	0.6697
Four medications at discharge^c^	**Normal weight**	1.0		1.0	
	**Overweight**	0.76 [0.56-1.03]^e^	0.0805	0.52 [0.34-0.80]^f^	0.0027
	**Obesity**	0.56 [0.37-0.86]^e^	0.0072	0.82 [0.54-1.26]^f^	0.3745
Less than four medications at discharge^d^	**Normal weight**	1.0		1.0	
	**Overweight**	0.67 [0.47-0.95]^e^	0.0260	1.21 [0.71-1.89]^f^	0.4095
	**Obesity**	0.70 [0.44-1.11]^e^	0.1320	1.03 [0.62-1.73]^f^	0.9062

HR=Hazard Ratio, CI=Confidence Interval, AMI=Acute Myocardial Infarction, BMI=Body Mass Index.

^a^Parsimonious model adjusted for sex, age, stroke, smoking, re-infarction, any reperfusion treatment (coronary artery bypass surgery, percutaneous coronary intervention (PCI) or thrombolysis), beta-blockers (medication prior AMI), all four medications at discharge (antiplatelet agents, beta-blockers, Angiotensin-converting enzyme inhibitors (ACEIs)/Angiotensin receptor blockers (ARBs), statins), left ventricular ejection fraction (<30% versus ≥ 30%).

^b^Parsimonious model adjusted for sex, age, hyperlipidemia, re-infarction, any reperfusion treatment, all four medications at discharge.

^c^Evidence-based medications (EBMs): Antiplatelet agents, beta-blockers, ACEIs/ARBs, statins.

^d^Either antiplatelet agents, beta-blockers, ACEIs/ARBs, statins or a combination of two or three of these medications.

^e^Parsimonious model adjusted for sex, age, stroke, smoking, re-infarction, any reperfusion treatment, beta-blockers (medication prior AMI), left ventricular ejection fraction (<30% versus ≥ 30%).

^f^Parsimonious model adjusted for sex, age, hyperlipidemia, re-infarction, any reperfusion treatment, statins (medication prior AMI).

## Discussion

In this population-based study we could show that an elevated BMI in AMI patients had significant effects on long-term survival in AMI patients without diabetes. In contrast, no such association could be detected in patients with diabetes. Interestingly, being prescribed statins prior AMI or four EBMs at hospital-discharge, overweight patients with diabetes appeared to have a significant survival benefit. Furthermore, in patients without diabetes the detected survival benefit of overweight and obesity attenuated with increasing observation time.

In line with our findings, Adamopoulos et al. [18] reported the absence of an obesity paradox among patients with diabetes and chronic heart failure. However, differences in study design and the fact that only two BMI groups (obese versus non-obese) were compared resulted in limited comparability to our study. Contrary to our findings, two studies showed that normal weight patients with diabetes and pre-existing cardiovascular events had the highest all-cause mortality rates compared to obese and overweight patients [16,17]. In addition, a study conducted in Japan compared 30-day survival rates following AMI of overweight (BMI ≥ 25 kg/m^2^) and normal weight (BMI < 25 kg/m^2^) patients with diabetes and found a significantly increased risk of dying among normal weight patients [24]. These contrasting results could derive from the very limited statistical power of the study as well as differences in study design. It has been shown that it can be misleading if BMI groups cover a wide range of values, since the association of BMI and mortality tends to follow a U- or J-shaped curve instead of increasing monotonically [25,26].

Our findings in patients without diabetes are in line with results of previous studies on patients with AMI [3-8,24,27-29] as well as recent systematic reviews including studies on patients with cardiovascular disease [30-32]. However, only one study has analyzed AMI patients without diabetes separately [18].

Interestingly, stratified analyses revealed a significant survival benefit in overweight patients with diabetes who were being prescribed statins prior AMI as well as in overweight patients with diabetes who received all four EBMs at hospital discharge. One study concluded that a statin therapy in patients with diabetes and without previous CVD might have a beneficial effect on survival [33] and other studies have shown that prescribing all four EBMs at hospital discharge resulted in a significant reduction in long-term morbidity and mortality [22,34]. It has been also demonstrated that medications prescribed to treat hypertension (beta-blockers, ACEIs, ARBs) and elevated cholesterol levels (statins) exert pleiotropic effects on myocardial remodeling and mortality after a cardiovascular event such as AMI [34].

In our study, the survival benefit of overweight and obese patients without diabetes attenuated with increasing observation time. Therefore, previous studies focusing on short-term mortality might have overestimated the protective effect of overweight and obesity on survival after AMI. The paradoxically protective effect could only be valid over a short period of time [35] and the fact that it attenuated with increasing observation time in our analysis could be a result of patients’ weight loss [17] or due to overweight and obese patients being more robust towards acute cardiovascular events.

Several factors could have caused the discrepancy in long-term survival in patients with and without diabetes: First, it can be hypothesized that an obesity paradox did not occur in patients with diabetes due to adverse effects of the disease or additional co-morbidities [36]. Combined with excess body weight they might be even more fatal. It has been shown that being overweight or obese does not exclude the possibility of being in a better cardiometabolic shape than normal weight patients [37]. A recent study demonstrated that normal weight patients with cardiometabolic dysfunction are at similar or even higher risk of cardiovascular morbidity and all-cause mortality in comparison with overweight and obese patients with cardiometabolic dysfunction [38]. However, in another study in patients with non-ST-segment elevation acute coronary syndrome only two components of the metabolic syndrome (low high-density lipoprotein and high triglycerides) were associated with higher 1-year mortality when examined separately [39]. Furthermore, it has been suggested to differentiate between metabolically healthy and metabolically obese patients within the BMI groups to better understand the effects of metabolic syndrome [37]. The cardiometabolic burden of adiposity, certain components of the metabolic syndrome and additional comorbidities might therefore play an important role in the explanation of the obesity paradox [37,38,40].

Second, we were not able to consider the time of diabetes diagnosis, the quality or stage of diabetes treatment as well as information on the treatment of overweight or obesity prior AMI. Previous studies revealed that the time of diabetes diagnosis might be relevant [41].

Third, additional comorbidities, such as cancer, changes in BMI as a result of weight gain or loss as well as adherence to prescribed medication after hospital discharge were not considered in our study. Recent findings indicate that BMI changes have an impact on long-term cardiovascular mortality [42].

Finally, there is evidence suggesting that the existence of an obesity paradox highly depends on the indices used to measure and classify normal weight, overweight and obesity [16,43,44]. Previous studies have shown that BMI combined with indices measuring the distribution of body-fat, such as waist-hip-ratio (WHR) or waist circumference (WC), offer more reliable results regarding mortality after cardiovascular events [16,45,46]. Especially in the context of CVDs, abdominal obesity is associated with a significantly increased mortality risk [47]. However, besides a higher adipose mass, overweight and obesity can be accompanied by an increase in lean mass, which might be, in turn, beneficial from a cardiometabolic perspective and could positively influence survival after cardiovascular events [8,37]. Moreover, abdominal obesity can also occur in normal weight people, which might increase their risk for cardiovascular events [37]. Using indices that better discriminate between fat and lean mass and that measure the distribution of body fat could help to shed light on this matter.

To our knowledge this is the first study investigating the association between BMI and long-term mortality in AMI patients that distinguished between patients with and without diabetes. This study has further strengths. First, important covariates such as medication prior and post AMI, in-hospital treatment and complications as well as a selection of comorbidities, were included. Second, we were able to examine the impact of diabetes and BMI on mortality over a longer period of time than any other study in this field before. Third, excluding patients with a BMI < 18.5 kg/m^2^ ensured that patients with possible cachexia caused by other diseases, such as cancer, were not included in our sample. Fourth, data were collected in the framework of a population-based registry with consecutive enrollment. This approach ensured that all patients with an AMI within the study area, who reached the hospital alive and survived longer than 28 days, were included in the sample. Finally, in contrast to other studies, patients were considered to suffer from diabetes if their condition was confirmed by chart-review.

Potential limitations of this study should be taken into account. Changes in body weight during the long follow-up period could not be considered. Patients older than 74 years were not included in our study. Due to a considerable number of patients who died before reaching the hospital or in the course of 28 days we cannot exclude possible bias of our results. Information on other relevant conditions affecting survival after AMI, such as any malignant disease and impaired renal function were not collected in this study. The proportion of patients with type-1-diabetes only accounted for 2.1% of the study population with diabetes mellitus (data not shown). We therefore believe that the potential bias lies within tolerable limits. Finally, no data was available on the duration of therapy or treatment prior and post AMI as well as on the patients’ compliance with taking prescribed medication before and after AMI had occurred.

## Conclusion

In AMI patients without diabetes we detected a significant protective effect of overweight and obesity on all-cause mortality, which attenuated with increasing observation time while remaining statistically significant. However, in AMI patients with diabetes being overweight or obese did not result in a survival benefit. Surprisingly, a paradoxical association was found in overweight AMI patients with diabetes who had received statins prior AMI and who had been prescribed with four EBMs at hospital discharge.

In order to thoroughly investigate the association between BMI and all-cause mortality in patients with and without diabetes further studies are needed.

## Additional files

Additional file 1:
**Further characteristics of patients with and without diabetes sub-divided into BMI groups.**
Additional file 2:
**Hazard ratios of overweight and obese patients with and without diabetes over increasing observation time.**
Additional file 3:
**Table supplementing Additional file** 2 **: Hazard ratios for mortality associated with elevated body mass index in patients with and without diabetes over increasing observation time.**

## Abbreviations

ACEI: Angiotensin-converting enzyme inhibitor
AMI: Acute myocardial infarction
ANOVA: Analysis of variance
ARB: Angiotensin receptor blocker
BMI: Body mass index
CI: Confidence interval
CVD: Cardiovascular disease
EBM: Evidence-based medication
HR: Hazard Ratio
IQR: Inter-quartile range
KORA: Cooperative Health Research in the Region of Augsburg
LVEF: Left ventricular ejection fraction
MI: Myocardial infarction
MONICA: Monitoring Trends and Determinants in Cardiovascular disease
NSTEMI: Non-ST-segment elevation myocardial infarction
PCI: Percutaneous coronary intervention
SD: Standard deviation
STEMI: ST-segment elevation myocardial infarction
VIF: Variance inflation factor
WC: Waist circumference
WHO: World Health Organization
WHR: Waist hip ratio
