# Supplementary figures and images for: Association of obesity and long-term mortality in patients with acute myocardial infarction with and without diabetes mellitus: results from the MONICA/KORA myocardial infarction registry
Source: Cardiovasc Diabetol. 2015 Feb 18;14:24. doi: 10.1186/s12933-015-0189-0 (PMC4396021; doi:10.1186/s12933-015-0189-0)

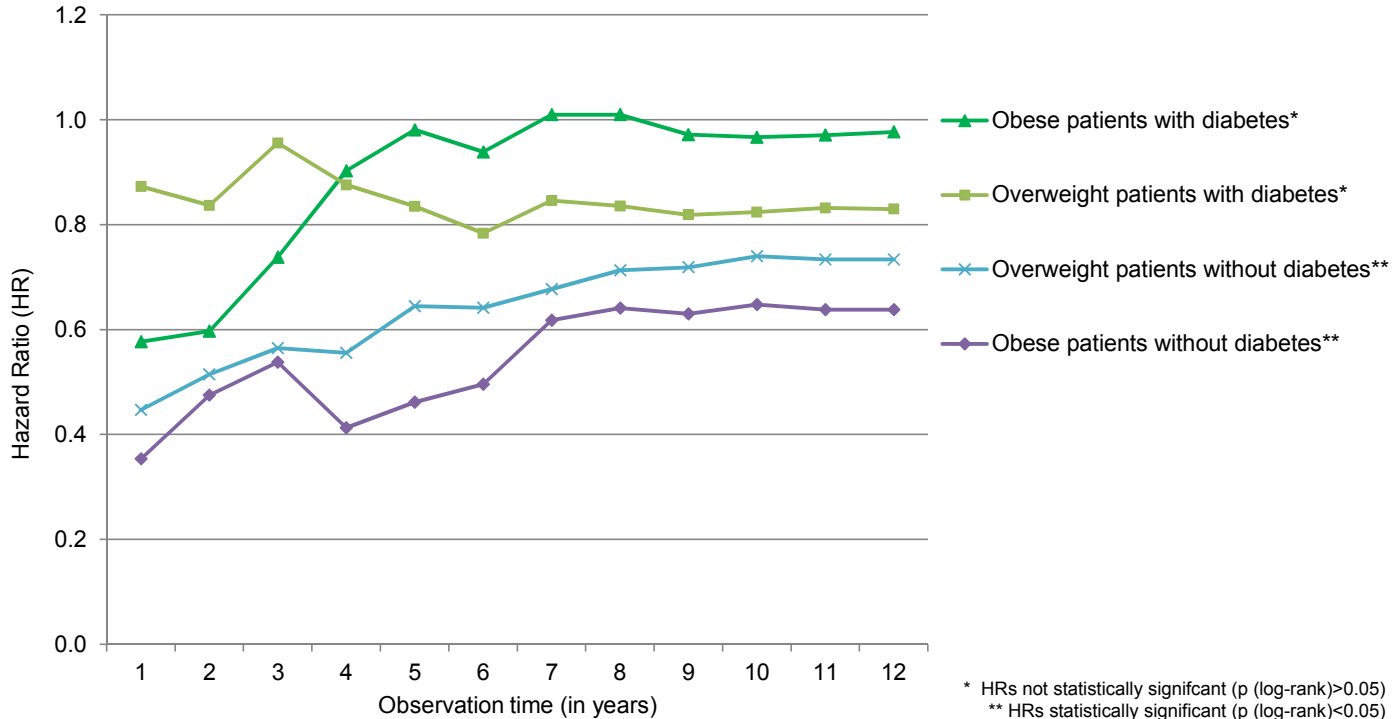

Supplement: Additional file 2: — Hazard ratios of overweight and obese patients with and without diabetes over increasing observation time. [file 12933_2015_189_MOESM2_ESM.pdf]
